# A Case of Metformin-Associated Lactic Acidosis Complicated by Acute Liver Failure, Acute Renal Failure, and Shock

**DOI:** 10.7759/cureus.61911

**Published:** 2024-06-07

**Authors:** Abdelhadi Farouji, Ahmad W Haddad, Mohammad Kloub, Amy Paige, Richard Miller

**Affiliations:** 1 Department of Internal Medicine, Saint Michael's Medical Center, New York Medical College, Newark, USA; 2 Department of Pulmonary and Critical Care Medicine, Saint Michael's Medical Center, New York Medical College, Newark, USA

**Keywords:** hemodialysis, high anion gap metabolic acidosis, alcoholic liver diseases, metformin-associated lactic acidosis, metformin

## Abstract

Metformin is an oral antihyperglycemic agent used for type 2 diabetes mellitus (T2DM) management and is considered to be the first-line treatment for diabetic patients. It works by improving insulin sensitivity, reducing intestinal absorption, and decreasing glucose production in the liver, leading to decreased blood glucose levels. It is generally considered a safe drug; however, it is associated with an uncommon but serious side effect known as metformin-associated lactic acidosis (MALA), a potentially life-threatening condition. Patients with renal failure and liver disease are at high risk of developing MALA; therefore, the medication should be used cautiously in these patients. The diagnosis of MALA requires high suspicion from the physician of this specific entity; otherwise, it may be easily missed. Herein, we report a case of a 63-year-old female with alcoholic liver disease on metformin who was found to have MALA complicated by acute decompensated liver failure, renal failure, and shock.

## Introduction

Metformin is a biguanide medication and is considered a first-line agent for type 2 diabetes mellitus (T2DM) management due to its beneficial effects on hemoglobin A1c, weight reduction, and cardiovascular mortality [1]. Also, it can be used in other conditions that are associated with insulin resistance and hyperinsulinemia such as polycystic ovarian syndrome (PCOS) [2]. The most common side effects associated with metformin are gastrointestinal symptoms such as nausea, vomiting, epigastric discomfort, and diarrhea; these symptoms have been reported in up to 30% of patients on metformin therapy [1]. However, metformin-associated lactic acidosis (MALA) is a rare but life-threatening adverse effect of metformin. It often requires intensive care support with a reported mortality rate of up to 50% [3]. The overall incidence of this complication is 1-9 cases per 100,000 patient-years, with most of the reported cases occurring in patients with compromised cardiac, pulmonary, hepatic, and renal functions [4]. Herein, we describe a 63-year-old patient with alcoholic liver disease on metformin treatment 500 mg twice daily who developed metformin-associated lactic acidosis complicated by acute liver failure, acute renal failure, and shock.

## Case presentation

A 63-year-old female was brought to the emergency department of Saint Michael's Medical Center, Newark, New Jersey, by her family as she was noticed to be confused over the past few hours. This confusion was associated with malaise, generalized weakness, diffuse abdominal pain, nausea and vomiting, jaundice, decreased oral intake, and decreased urine output over the past day. She had a past medical history of type 2 diabetes mellitus (T2DM), hypertension, hyperlipidemia, and alcoholic liver disease (stage 2). She is an active drinker, drinking around two to three 12-ounce beers daily. Her ongoing medications include metformin 500 mg twice daily, amlodipine 5 mg daily, and atorvastatin 20 mg daily.

Vital signs on initial presentation revealed a heart rate of 115 beats per minute, blood pressure of 101/65 mmHg, temperature of 36.6°C, respiratory rate of 28 breaths per minute, and oxygen saturation of 97% on room air. On physical examination, she was alert and oriented to herself only. Head and neck examination revealed scleral icterus and dry mucous membranes; skin examination revealed diffuse jaundice and decreased skin turgor. The abdomen was distended with mild tenderness to palpation in epigastric and right upper quadrant regions with hepatomegaly; also, asterixis was noticed. The remainder of her physical examination was unremarkable.

Initial laboratory tests revealed a pH of 7.34, critically low bicarbonate level, high anion gap metabolic acidosis, highly elevated lactic acid and ammonia, elevated aspartate transaminase (AST) and alanine transaminase (ALT), and high international normalized ratio (INR) (Table 1). Serum glucose was 85 mg/dL on presentation. Her urinalysis was negative for ketones, nitrates, and leukocyte esterase. Urine drug screen (UDS) was negative. Serum acetaminophen and salicylate levels were within normal limits. Computed tomography (CT) of the head showed no acute intracranial process, CT of the abdomen showed hepatomegaly with diffuse fatty infiltration, and no calcified gallstones or pericholecystic inflammation were seen. Vascular Doppler ultrasound of the liver did not show any signs of portal vein thrombosis.

**Table 1:** Laboratory tests on admission and after the hemodialysis BUN: blood urea nitrogen, AST: aspartate transaminase, ALT: alanine transaminase, CK: creatine kinase, PTT: partial thromboplastin time, PT: prothrombin time, INR: international normalized ratio, WBC: white blood cell

Laboratory test	Admission laboratory results	Laboratory results after hemodialysis	Reference range
Blood chemistry			
Sodium	142 mmol/L	144 mmol/L	136-145 mmol/L
Potassium	2.2 mmol/L	3.1 mmol/L	3.5-5.3 mmol/L
Bicarbonate	8.1 mmol/L	23.5 mmol/L	20-31 mmol/L
Chloride	98 mmol/L	105 mmol/L	98-110 mmol/L
Anion gap	36 mmol/L	15 mmol/L	<12 mmol/L
BUN	15 mg/dL	5 mg/dL	6-24 mg/dL
Creatinine	1.5 mg/dL	0.5 mg/dL	0.6-1.2 mg/dL
Lactic acid	22.4 mmol/L	13 mmol/L	0-2 mmol/L
Total bilirubin	15.2 mg/dL	11.7 mg/dL	0.2-1.2 mg/dL
Direct bilirubin	10.60 mg/dL	-	0-0.3 mg/dL
AST	1,243 U/L	705 U/L	10-36 U/L
ALT	159 U/L	120 U/L	9-46 U/L
Alkaline phosphatase	474 U/L	312 U/L	40-115 U/L
Total CK	120 U/L	-	38-176 U/L
Ammonia	127 µmol/L	-	11-32 µmol/L
Acetaminophen level	4.6 µg/mL	-	0-20 µg/mL
Hepatitis A IgM antibody	Non-reactive	-	-
Hepatitis B surface antigen	Non-reactive	-	-
Hepatitis B core IgM antibody	Non-reactive	-	-
Hepatitis C antibody	Non-reactive	-	-
PTT	45.2 seconds	-	26-36 seconds
PT	37.9 seconds	-	10-12 seconds
INR	3.16	-	0.91-1.1
Complete blood count	-	-	-
WBC	15.3 × 10^3^/µL	12.3 × 10^3^/µL	4.4-11 × 10^3^/µL
Hemoglobin	8.5 g/dL	6.5 g/dL	13.5-17.5 g/dL
Platelet	218 × 10^3^/µL	121 × 10^3^/µL	150-450 × 10^3^/µL

The patient was admitted to the medical intensive care unit (MICU) and started on aggressive IV fluid resuscitation. Lactulose and rifaximin were started because of hepatic encephalopathy. She was started on antimicrobial therapy including vancomycin and meropenem after blood and urine cultures were taken. Then, she became hypotensive, requiring the use of vasopressors. The patient's clinical presentation was highly suspicious of MALA because of high anion gap metabolic acidosis and highly elevated lactic acid. Acetaminophen level and salicylate level were within normal limits, and urine drug screen and viral hepatitis serologies were negative. Therefore, nephrology was consulted and recommended an emergent hemodialysis. An emergent hemodialysis for three hours was completed, which resulted in a rapid improvement in her lactic acidosis and liver function tests (Table 1). The patient's antibiotics were discontinued after negative blood and urine cultures.

## Discussion

Being one of the oldest antidiabetic medications and one of the most commonly prescribed drugs for type 2 diabetes mellitus nowadays, metformin has been associated with some mild side effects including most commonly nausea, a metallic taste, and diarrhea [1]. Throughout our paper, we will be reporting a case of one of the rare and most severe side effects associated with metformin known as metformin-associated lactic acidosis (MALA).

In view of being mostly a diagnosis of exclusion, with a low incidence of around <10 cases per 100,000 patient-years, MALA must always be kept in mind in patients using metformin since it has been linked to a very high mortality rate of up to 50% [3]. Recognition is therefore very important and crucial for proper emergent management, which is mostly with hemodialysis. Moreover, clues that usually point toward MALA include very elevated levels of lactic acid leading to unmeasured anions and a very high anion gap [3].

The mechanism of MALA is complex; lactate can either be oxidized to carbon dioxide and water by mitochondria to generate energy or converted back to glucose by the gluconeogenesis process in the liver and kidney. Lactic acidosis occurs during conditions of excessive lactate production and/or impaired hepatic lactate removal [5]. Metformin promotes lactate production by promoting the conversion of glucose to lactate in the splanchnic bed of the small intestine. Also, metformin inhibits mitochondrial respiratory chain complex 1, leading to decreased hepatic gluconeogenesis and lactate accumulation [5].

Metformin is already known to be cautiously used in patients with chronic kidney disease since it can lead to the accumulation of lactic acid by preventing drug excretion, in addition to also being used cautiously in patients with congestive heart failure and chronic liver disease, which could also lead to lactate accumulation [6]. According to García-Compeán et al., the prevalence of diabetes mellitus in liver cirrhosis is about 30% [7]. Furthermore, since the liver is known to be highly involved in gluconeogenesis, cirrhotic patients in whom metformin is prescribed are therefore more prone to suffer from dramatically elevated lactic acid since lactate removal by the liver is affected. It is thus why, in addition to patients with chronic kidney disease being discouraged from using metformin, patients with chronic liver disease have also been recommended sometimes to avoid the use of metformin [6]. Although the evidence of preventing patients with chronic liver disease from using metformin is very scarce and is mostly limited to case reports and patients with active alcohol use, it must be kept in mind that there is also, on the other hand, very dense literature concerning the favorable effects of biguanides in patients with liver disease, specifically in non-alcoholic fatty liver disease patients [8].

Clinical symptoms of MALA on presentation are non-specific, including nausea, vomiting, and epigastric pain. Most patients with MALA are hypotensive and develop circulatory shock on presentation or shortly after. Also, hypoxic respiratory failure requiring mechanical ventilation has been reported in the literature [9].

The differential diagnosis for MALA includes other causes of hyperlactatemia and metabolic acidosis. These causes can be classified into toxicological and non-toxicological etiologies [4]. Non-toxicological etiologies include sepsis, shock, status epilepticus, liver failure, mesenteric ischemia, and diabetic and alcoholic ketoacidosis [4]. Other toxicological etiologies may include cellular asphyxiants, uncoupling agents, impaired hepatic clearance of lactate, and overdoses resulting in seizures. Examples include cyanide, isoniazid, antiretroviral drugs, linezolid, propylene glycol, salicylate, propofol infusion syndrome, and massive acetaminophen overdose [4].

The management of metformin-associated metabolic acidosis should be aggressive. This aggressive management strategy includes supportive care, treatment of underlying disease, correction of acidemia, acceleration of lactate metabolism, and elimination of the offending drug by renal excretion or dialysis [10]. Patients with MALA may exhibit signs of shock requiring aggressive management, including intubation, ventilation, and vasopressor support [4]. Sodium bicarbonate infusion is recommended for pH < 7.20, especially in the setting of underlying cardiovascular disease or hemodynamic compromise [4].

Metformin has a small molecular weight and lacks any significant protein binding feature, so it can be easily removed from the bloodstream by hemodialysis or continuous renal replacement therapy. Therefore, renal replacement therapy is the main treatment of MALA. Dialysis techniques not only remove the metformin from the bloodstream but also correct rapidly the acid-base disorders [10]. Hemodialysis should be initiated for pH < 7.0 and lactate > 20, and in patients who do not respond to conservative therapies [4]. Intermittent hemodialysis appears to be more effective at clearing metformin and lactate than continuous clearance modalities and is preferred with concurrent use of vasopressors [4]. Extracorporeal membrane oxygenation (ECMO), when available, is a potential intervention if hemodynamics does not allow for hemodialysis and if unresponsive to vasopressors [4].

In patients with MALA secondary to underlying disease, mortality correlates with underlying acute or comorbid conditions. However, it is not related to plasma metformin levels or arterial lactate levels [11]. On the other hand, in patients with MALA from confirmed acute metformin overdose, mortality correlates with lower serum pH and higher serum lactate levels [12].

We described a case of a 63-year-old female patient with alcoholic liver disease on metformin treatment who developed metformin-associated lactic acidosis complicated by acute liver failure, acute renal failure, and shock, which was managed by urgent hemodialysis. This case increases the awareness of this serious and fatal complication of metformin therapy, especially in patients with underlying liver disease or other risk factors.

## Conclusions

It is therefore our goal to state the importance of having MALA high on the differential in a patient with a history of liver disease, specifically alcoholic liver disease presenting with a very elevated lactic acidosis and anion gap, and being knowledgeable that prompt hemodialysis could be lifesaving. Regardless of our paper, one must keep in mind that the efficacy of metformin has been greatly established for many decades and that the threshold to not use it in patients with liver disease is encouraged to be low and individualized depending on each patient's case and comorbidities to avoid preventing a majority of patients from benefiting from an overall benign antidiabetic medication.
